# Three-dimensional quantification of twisting in the Arabidopsis petiole

**DOI:** 10.1007/s10265-021-01291-7

**Published:** 2021-04-11

**Authors:** Yuta Otsuka, Hirokazu Tsukaya

**Affiliations:** Department of Biological Sciences, Graduate School of Science, The University of Tokyo, 7-3-1, Hongo, Bunkyo-ku, Tokyo, 113-0033 Japan

**Keywords:** 3D, *Arabidopsis thaliana* (Arabidopsis), Imaging, Leaf, Phototropism, Twist

## Abstract

Organisms have a variety of three-dimensional (3D) structures that change over time. These changes include twisting, which is 3D deformation that cannot happen in two dimensions. Twisting is linked to important adaptive functions of organs, such as adjusting the orientation of leaves and flowers in plants to align with environmental stimuli (e.g. light, gravity). Despite its importance, the underlying mechanism for twisting remains to be determined, partly because there is no rigorous method for quantifying the twisting of plant organs. Conventional studies have relied on approximate measurements of the twisting angle in 2D, with arbitrary choices of observation angle. Here, we present the first rigorous quantification of the 3D twisting angles of Arabidopsis petioles based on light sheet microscopy. Mathematical separation of bending and twisting with strict definition of petiole cross-sections were implemented; differences in the spatial distribution of bending and twisting were detected via the quantification of angles along the petiole. Based on the measured values, we discuss that minute degrees of differential growth can result in pronounced twisting in petioles.

**Supplementary Information:**

The online version contains supplementary material available at 10.1007/s10265-021-01291-7.

## Introduction

Organisms exhibit three-dimensional (3D) morphology, which may change over time. The entire morphology of an organ was captured and analyzed using quantitative methods, such as geometric morphometrics (Bouby et al. 2020; Savriama 2018). However, such methods are not sufficient to examine the function of an organ in detail; measurement of specific morphological parameters is also required.

**Fig. 1:**
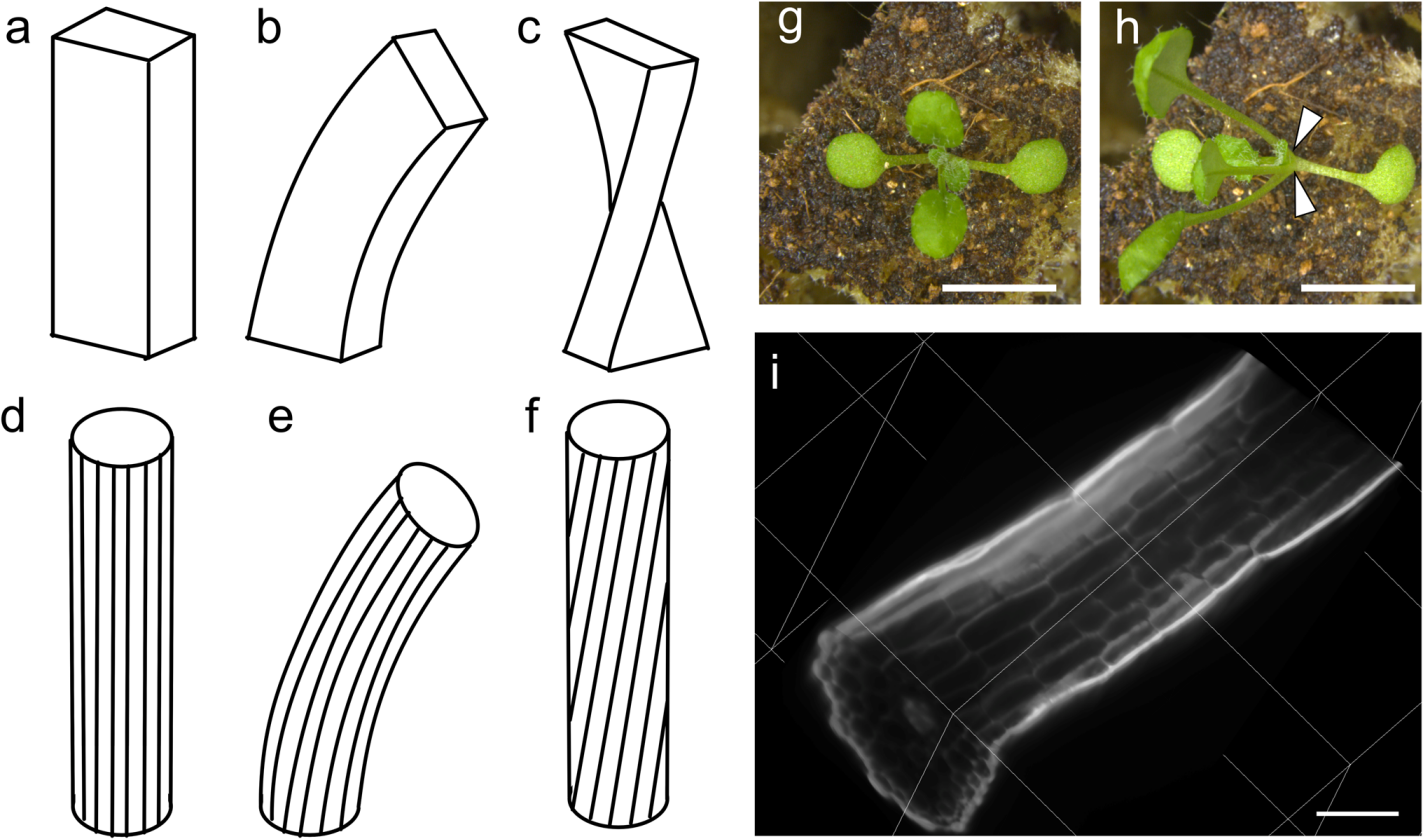
Twisting and bending are different components of petiole deformation. The straight pre-deformed box (**a**), bent box (**b**), and twisted box (**c**) show the differences between twisting and bending. The bent box (**b**) exhibits curvature of the long axis of the box. The twisted box (**c**) maintains a straight long axis with rotation of a cross-section around the long axis. In radially symmetric organ (**d**–**f**), bending (**e**) and twisting (**f**) can be observed following epidermal cell files shown as bent (**e**) or inclined (**f**) lines. The first pair of leaves of Arabidopsis grown under normal symmetric light irradiation at 12 days after sowing (**g**) have straight pre-deformed petioles resembling the straight box in (**a**). After 3 days of lateral light irradiation (from the left of the photograph), petioles bent at the base (arrowheads) and twisted to turn the adaxial side of the leaf blade toward the light source (**h**). **i** 3D image of a petiole reconstructed from light sheet microscopy images, which have been cut virtually in middle to show inner cells. Bars 5 mm (**g**, **h**), 100 μm (**i**)

Plants have colonized land based on their ability to modify their 3D morphologies in response to environmental stimuli. They may be analyzed via both holistic approaches and specific measurements. One of the principal examples analyzed using a specific approach is phototropism, the movement of plant organs in a direction specified by an external environmental stimulus (Darwin and Darwin 1880; Moulton et al. 2020). Phototropism in radially symmetric organs (e.g. the etiolated hypocotyl and root of *Arabidopsis thaliana,* Arabidopsis hereafter) is typically caused by bending of an organ (Fig. 1d, e). Bending is the result of differential growth on two sides of an organ. In phototropism of the Arabidopsis hypocotyl, the shaded side elongates more than the irradiated side (Friml et al. 2002). The hypocotyl then bends to resolve the tension created by differential growth. The ratio of the length of the shaded side to that of the irradiated side is approximately 1.1:1 in 3-mm segments bent at 90° (Friml et al. 2002).

In addition to bending (Fig. 1a, b), dorsoventral organs (e.g. leaves) undergo twisting (Fig. 1a, c), another component of phototropism (Iino 2001; Koller 1990). Twisting is not the deformation of the midline of an organ but rather, it is the rotation of a cross-section around the axis of the midline (Fig. 1a, c). Stimulus-directed twisting has been referred to by a number of terms, including strophism (Snow 1962), heliotropism (Iino 2001), resupination (Dines and Bell 1994; Harley et al. 2017), and torsion (Borchers et al. 2018), with slightly different implications. Here, we use the word “twist” as a simple expression of the deformation represented in Fig. 1c. Twisting can rotate the dorsoventral axis of an organ to achieve a certain angle relative to the directing stimulus (e.g. light, gravity). Light-directed twisting of plant organs often has clear adaptive benefits. Twisting of the basal part of the leaf adjusts the orientation of the adaxial surface of the lamina to face the light source, enhancing photosynthesis (Koller 1990; Terashima and Saeki 1985). Flower orientation is also adjusted by twisting to attract specific pollinators (Darwin and Darwin 1880; Harley et al. 2017).

In accordance with its ecological significance, twisting has been recognized by researchers as a distinct component of phototropism and is studied independently of bending (Borchers et al. 2018; Darwin and Darwin 1880; Snow 1962). However, the measurement of twisting was performed approximately in 2D, and did not strictly exclude potential bias resulting from the bending of organs and/or the oblique angle of observation views (Borchers et al. 2018; Buschmann et al. 2009; Snow 1962; Thitamadee et al. 2002). Although these rough methods are sufficient for detecting large differences in twisting angles among samples, they are not suitable for characterizing local twisting angles or the spatial distribution of twisting along the organ. Understanding this spatial distribution is necessary to examine the roles of molecules that are essential for organ twisting. Thus, concrete quantification methods to evaluate the local twisting angle at different parts of an organ are needed. Recently, theoretical approaches for simulating plant tropisms with mathematical definitions of bending and twisting have been reported (Moulton et al. 2020). In these studies, theoretical predictions and experimental results for bending in response to multiple environmental stimuli were compared. However, in the case of twisting, theoretical predictions have not yet been compared to experimental measurements, possibly due to the lack of appropriate measurement methods. As a result, bending is now relatively well-understood, whereas the mechanisms for twisting remain unclear.

In addition to the twisting in photoresponse, there are variety of twisting in plants also in radially symmetric organs (Fig. 1d, f) (reviewed in Buschmann and Borchers 2020; Nakamura and Hashimoto 2020; Smyth 2016). Those twisted growths sometimes are associated with the spiral growth of organ (e.g. in winding vines) and referred together as helical growth (Buschmann and Borchers 2020; Smyth 2016). However, spiral growth and twisting are distinct deformation. Spiral is a form of curve of organ midline (Smyth 2016). In contrast, twisting is a rotation of cross-section around midline and is not the curve of midline (Smyth 2016; Snow 1962).

In the case of twisting of microtubule-related mutants, cellular and molecular basis is progressively being revealed (Buschmann et al. 2009; Nakamura and Hashimoto 2020; Thitamadee et al. 2002), but detailed analysis on spatial distribution of the twisting is lacking. Similarly, also in other mutants (Geisler et al. 2003; Saffer et al. 2017) and other naturally occurring twisting (Abraham and Elbaum 2013; Darwin and Darwin 1880; Smyth 2016), quantification of twisting distribution would help further understanding of varieties of underlying mechanisms.

Here, we present a method to quantify 3D bending and twisting. Using this new method, we characterized the spatial distribution of local twisting angles along light-twisted Arabidopsis petioles. Furthermore, based on the measured values, we present a geometric estimation and discuss that minute differential growth can result in large twists in petioles.

## Materials and methods

### Plant growth conditions

*Arabidopsis thaliana* (accession Columbia) was used throughout this study. As a growth medium, rockwool (M35T40, Nittobo) was washed once with tap water and divided into quarters. Seeds were sown on quarter-sized blocks and kept at 4 ℃ for a 2-day stratification period. The seeds were germinated in growth chambers with 50 µmol m^−2^ s^−1^ white light at 22 ℃. The rockwool was covered with the powder of crushed Golden Pete Bann (Sakata) at 1 day after germination and kept wet by a supply of 0.5 g L^–1^ Hyponex water (Hyponex Japan).

At 11 or 12 days after sowing, plants were placed on a tray to align the first pair of leaves in a particular direction. Then, the plants were moved to a chamber in a dark room with lateral blue light illumination from a 5 µmol m^−2^ s^−1^ LED-mB lighting unit (peak wavelength: 470 nm, EYELA) and red illumination from above from a 25 µmol m^−2^ s^−1^ LED-mR lighting unit (peak wavelength: 660 nm, EYELA) or an ISL-150 × 150-H4RR lighting unit (peak wavelength: 660 nm, CCS).

### Light sheet microscopy

Plants were harvested with forceps holding the hypocotyl, so as not to damage the petiole of the first pair of leaves. A cotyledon on a particular side was cut to mark the orientation of the blue light. Plants were fixed in 4% (w/v) paraformaldehyde (PFA; Wako) with 0.1% (v/v) Tween-20 in 1X phosphate buffered saline (PBS; pH 7.4), with repeated − 90 kPa degassing until the entire plant sank. After 1 day of fixation at 4 ℃, the plants were washed twice in 1X PBS and treated with ClearSee solution (Kurihara et al. 2015) at 4 ℃ for more than one week. The treated leaves were stained with 10 mg L^-1^ Calcofluor White M2R or 0.1% (w/v) Direct Red 23.

The petiole of a leaf from the first pair was separated from the blade with a fine razor blade. To avoid damage to the petiole, only young primordia and laminae were held by forceps. Next, petioles were transferred to 1% (w/v) low-melting-point agarose (Promega) kept at 40 ℃. Then, the petioles were carefully transferred to glass capillaries filled with low-melting-point agarose. After the gel had cooled sufficiently, the samples were placed in the water chamber of a Z.1 light sheet microscope (Zeiss). Calcofluor M2R and Direct Red 23 were excited with 405-nm and 561-nm lasers, respectively. Images representing both sides of the dual-side illumination were merged using the “mean” mode.

## Results

### Establishment of a 3D measurement method for the Arabidopsis petiole

We used Arabidopsis petioles as a model material to demonstrate our method for quantifying the twisting of plant organs. Our choice of a frequently used model species enables the future combination of our method with genetic and molecular biology tools. The first leaves at the 11th or 12th day after sowing (Fig. 1g) were irradiated with blue light from the side, which induces twisting in Arabidopsis petioles (Inoue et al. 2008). As reported previously, we monitored the twisting of the petioles so as to orient the blades perpendicular to the light direction (Fig. 1h).

To measure twisting, we first needed to capture the 3D structure of the twisted petioles. There were several options for this type of measurement: photogrammetry, micro-X-ray computed tomography (CT), and various kinds of laser microscopy. Photogrammetry has been widely used for plant phenotyping, including the measurement of leaf length and area (An et al. 2016). However, the resolution is typically not high enough to capture the twisting of petioles, which requires resolution on the order of 10 µm (An et al. 2016). Micro-X-ray CT scanning is applicable to plant organs (Karahara et al. 2015) but is incompatible with most molecular biological techniques, including the use of fluorescent protein reporters. Thus, considering the need for subcellular resolution and future applicability, we chose laser microscopy to capture the 3D shapes of the petioles. There are several types of laser microscopy. We employed light sheet microscopy, which allows fast scanning and efficient illumination with a sheet of light (Ovečka et al. 2018). The following procedure may also be adapted for images acquired with conventional confocal laser scanning microscopy, although special considerations will be required, such as sample embedding in a gel to avoid deformation of petioles by gravity and the use of long-working-distance lenses to capture non-flat petioles.

We first established a sample preparation procedure for light sheet microscopy. We tried several sizes of centrifuge tubes for fixing petioles with 4% PFA and found that 25-mL tubes were large enough to hold 2-week-old Arabidopsis samples without exposing them to excessive external force. We noted internal cracks in tissues when petioles were fixed with excessively rapid increases in pressure. Thus, we fixed petioles with as gradual a pressure gradient as possible (approximately 5 kPa min^–1^) to avoid external forces. Next, petioles were optically cleared with ClearSee reagent (Kurihara et al. 2015). We used 10 mg L^-1^ Calcofluor M2R and 0.1% (w/v) Direct Red 23 (Ursache et al. 2018) to stain cell walls, and both clearly enabled the visualization of cell shape, thus allowing for future combination with variety of fluorescent reporters.

To set optically cleared petioles in the water chamber of a light sheet microscope, we first used fluorinated ethylene propylene (FEP) tubes filled with ClearSee reagent. However, possibly owing to a mismatch of refractive indices between ClearSee reagent (*n* = 1.41) and FEP tubes and water (*n* = 1.34 and 1.33, respectively), the images taken with left and right illumination were skewed differently, hindering the dual-side imposition of the two images. Thus, we tried to minimize high-refractive-index regions by replacing ClearSee solution and FEP tubes with 1% low-melting-point agarose. Using this approach, we successfully acquired z-stack images of the 3D structure of the entire petiole, except for the most basal part (approximately 200 µm from the shoot apex) that was obscured by other leaves and primordia (Fig. 1i).

Next, we established our in-house program to calculate the angles of bending and twisting from the light sheet microscope images. The mathematics to separately describe the bending and twisting of rod-shaped object have previously been employed in the field of mechanics (Audoly and Pomeau 2010). Recently, these mathematics have been applied to computer simulations of phototropism (Moulton et al. 2020).

**Fig. 2:**
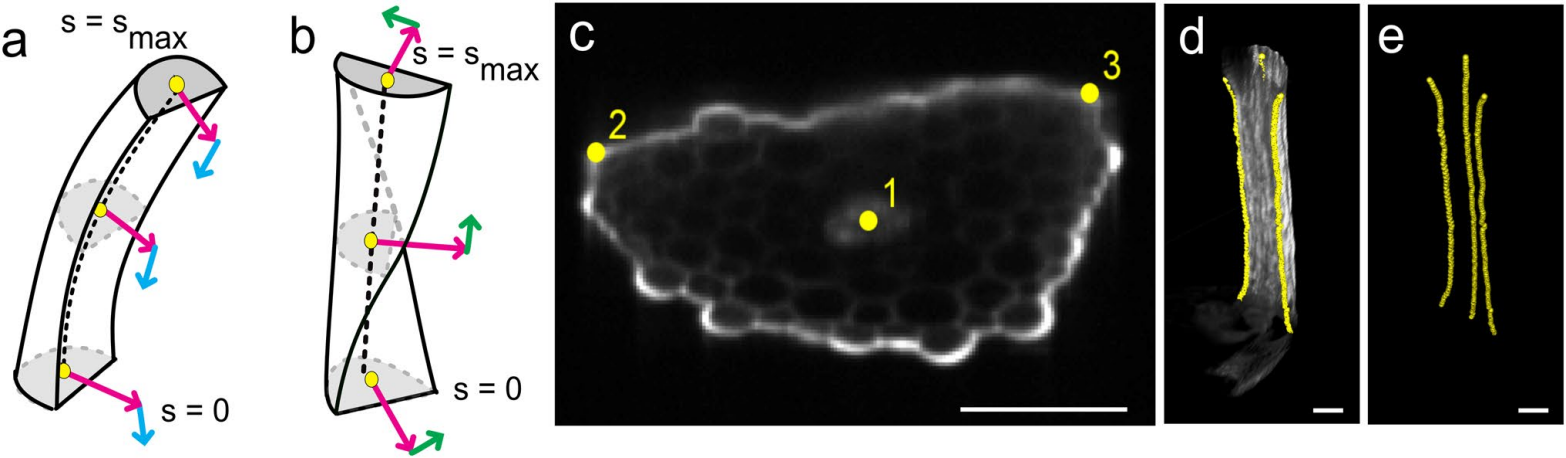
Mathematical dissection of twisting and bending. **a**, **b** *s* indicates the position along the petiole. Bending and twisting is measured by changes of the e_adaxial_(s), the adaxial–abaxial vector (magenta arrow) with increasing *s*. The cyan arrow (**a**) indicates changes of e_adaxial_ that is tangent to the midline, which corresponds to vertical bending. Lateral bending is not indicated with an arrow but consisted of changes in the midline direction perpendicular to e_adaxial_. Twisting is indicated by green arrow (**b**), which represents changes of e_adaxial_ in a direction perpendicular to the midline of the petiole. **c** An oblique approximate cross section of an Arabidopsis petiole as observed in the MTrackJ window in ImageJ. The yellow circles indicate tracked points. Circle #1 indicate the tracked midvein of the petiole, which is used as the midline. Circles #2, 3 indicate the tracked margins of the petiole, which are used to define e_adaxial_. **d**, **e** Tracked points are shown as yellow balls in 3D. Petiole cell walls are shown in gray in **d** but not in **e**. Bars 100 μm

We adopted the mathematical definitions of Moulton et al. (2020) for twisting and bending angles (Fig. 2a, b) to calculate the experimentally observed twisting of plant organs. Twisting (green arrow in Fig. 2b) is defined as the rotation of the adaxial–abaxial vector (magenta arrow in Fig. 2a, b) around the midline axis of the petiole. In other words, twisting is a rotation of the cross section around axis of organ midline (Fig. 2b). Vertical bending (cyan arrow in Fig. 2a) is defined as the rotation of the adaxial–abaxial vector in the plane perpendicular to the lateral direction; in other words, the rotation of a cross-section around medio-lateral axis (Fig. 2a). Similarly, bending in a direction perpendicular to the vertical axis is defined as lateral bending.

To apply the above definitions to the actual plant images, we first needed 3D coordinates of the midline and adaxial–abaxial vector. We used central vasculature as the midline and two margins of the petiole to define the adaxial–abaxial vector (Fig. 2c–e). We used the MTrackJ plugin in ImageJ to obtain the 3D coordinates of each curve (the midline and both margins). The margins of petiole are found based on the curvature of the epidermal layer in the x–y plane section. More precisely, we tracked the point in which approximately flat adaxial surface of petiole ends with major curvature as petiole margin. Vasculature is tracked as the center of brightest spot in the middle of x–y section of petiole. The tracking required intensive amount of point selection by hand. Thus, rather than using mouse-click tracking, we used iPad (Apple Inc.) mirror screening and an Apple Pencil (Apple Inc.), which enabled accurate and fast tracking with less damage to the hand of a researcher.

**Fig. 3:**
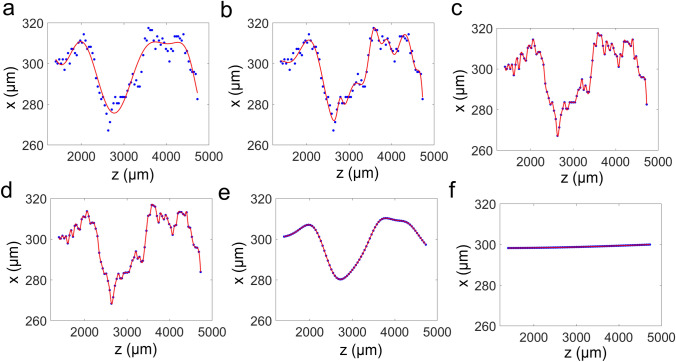
Comparison of smoothing methods to obtain continuous curves. Tracked (x, z) coordinates of the petiole margin (blue dots) were converted to continuous curves (red line) with ninth-polynomial fitting (**a**), 8-Gaussian fitting (**b**), or cubic interpolation (**c**). Note that cubic interpolation provided the best match to the original tracked data, indicating the complicated high-degree-of-freedom curvature of the petiole margin in the x–z plane. Smoothing of the x–z plot with a 100-μm Gaussian filter (**d**) had a minimal effect in reducing noise, whereas smoothing with a 10,000-μm Gaussian filter (**f**) resulted in the loss of information, leading to an overly simplistic curve. A 1,000-μm filter (**e**) successfully removed noise while maintaining the shape of the original plot

The tracked points were then converted to smooth continuous curves to calculate derivatives. We tried several methods of function-fitting: ninth-polynomial fitting, 8-Gaussian fitting (fitting with a combination of eight Gaussian curves), and cubic interpolation. Polynomial fitting and Gaussian fitting resulted in misfit of the specific form of the chosen fitting function (Fig. 3a, b), possibly due to the low degree of freedom. By contrast, cubic interpolation resulted in no such misfit (Fig. 3c). However, noise from manual tracking and inherent bumpiness of petiole remained after cubic interpolation. Thus, we denoised the tracked points with Gaussian filters before cubic interpolation. We tried several window sizes for Gaussian smoothing. Excessively narrow windows resulted in no effective smoothing (Fig. 3d), whereas excessively broad windows eliminated information by over-smoothing (Fig. 3f). Considering this trade-off, we chose an optimal window size of 1,000 µm (Fig. 3e).

To obtain derivatives, in addition to the smoothened continuous curves, we needed the parameter representing the position along the petiole. Because petioles were not strictly vertical to any axis in the z-stack images, we did not use x, y, z coordinates to indicate the “position along the petiole”. Instead, we calculated the curved distance from the blade-petiole junction along the center curve and used it as the “position along the petiole” (= s (z)), as follows:

$${\text{s}}\left( \zeta \right) = { }\mathop \smallint \limits_{0}^{\zeta } \sqrt {{\text{x}}_{{{\text{midline}}}}^{2} + {\text{y}}_{{{\text{midline}}}}^{2} + {\text{ z}}_{{{\text{midline}}}}^{2} } {\text{dz}}_{{{\text{midline}}}} .$$

Then, the cross section was defined as a plane perpendicular to the midline. At each position *s*, the unique cross point of the cubic-interpolated curves and the cross section could be found, because the longitudinal direction of the petiole was nearly parallel to the z direction. The 3D coordinates of the cross point were re-defined as x _left margin_(s) and x _right margin_(s).

Next, we obtained unit vectors in the medio-lateral, longitudinal, and adaxial–abaxial directions (same direction as green, cyan, magenta arrows in Fig. 2a, b, respectively) defined as follows:

$${\text{e}}_{{{\text{lateral}}}} \left( {\text{s}} \right) = {\text{unit vector in the same direction as }}\left( {{\text{x}}_{{\text{left margin}}} \left( {\text{s}} \right) - {\text{ x}}_{{\text{right margin}}} \left( {\text{s}} \right)} \right)$$

$${\text{e}}_{{{\text{longitudinal}}}} \left( {\text{s}} \right) = {\text{unit vector in the same direction as }}\left( {\frac{{{\text{dx}}_{{{\text{midline}}}} }}{{{\text{ds}}}}\left( {\text{s}} \right)} \right)$$

$${\text{e}}_{{{\text{adaxial}}}} \left( {\text{s}} \right) = {\text{ e}}_{{{\text{longitudinal}}}} \left( {\text{s}} \right) \times {\text{e}}_{{{\text{lateral}}}} \left( {\text{s}} \right),$$

in which “$\times$” refers to cross product of vectors.

Using s-derivatives of the unit vectors, local angles of twisting and bending were calculated as follows, in which “$\cdot$” refers to dot product of vectors:

$${\text{twisting }}\left[ {{\text{rad}}/{\text{length}}} \right]{ } = \tau \left( {\text{s}} \right) = { }\frac{{{\text{de}}_{{{\text{adaxial}}}} }}{{{\text{ds}}}} \cdot {\text{e}}_{{{\text{lateral}}}}$$

$${\text{lateral bending }}\left[ {{\text{rad}}/{\text{length}}} \right] = \kappa 1\left( {\text{s}} \right) = { }\frac{{{\text{de}}_{{{\text{longitudinal}}}} }}{{{\text{ds}}}} \cdot {\text{e}}_{{{\text{lateral}}}}$$

$${\text{vertical bending }}\left[ {{\text{rad}}/{\text{length}}} \right] = \kappa 2\left( {\text{s}} \right) = { }\frac{{{\text{de}}_{{{\text{adaxial}}}} }}{{{\text{ds}}}} \cdot {\text{e}}_{{{\text{longitudinal}}}} .$$

Cumulative angles were calculated as the integration of local angles from blade-petiole junctions. All calculations were programmed as in-house MATLAB code and executed within several minutes on a laptop.

Taken together, we established a concrete procedure to measure the 3D twisting angles of petioles.

### Quantified spatial distribution of twisting and bending

**Fig. 4:**
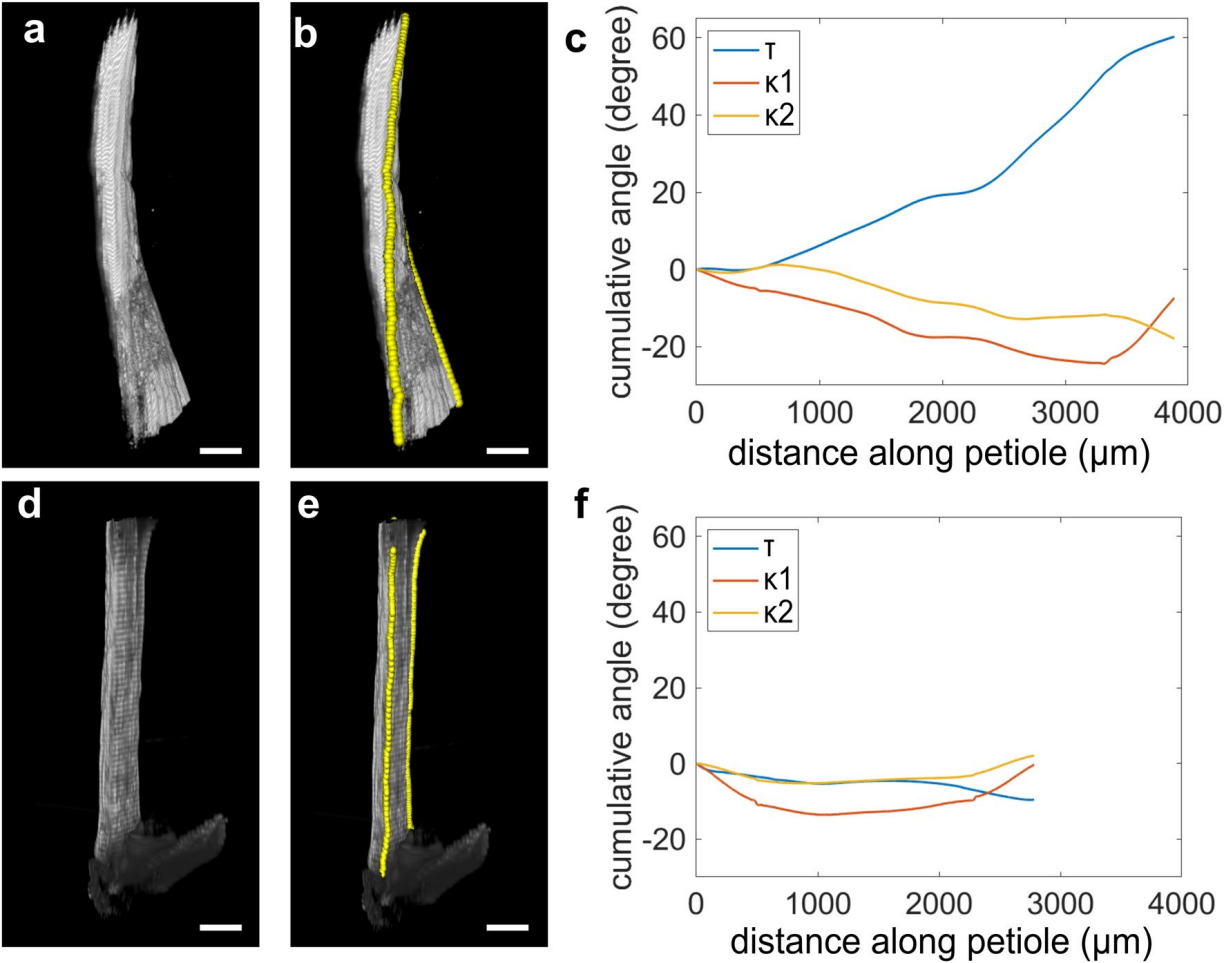
Quantified spatial distribution of twisting and bending. Petiole irradiated with lateral blue light for 3 days are imaged in light sheet microscope (**a**). 3D coordinates of margins of petiole shown as yellow balls (**b**) were used to calculate cumulative angles of twisting (blue), lateral bending (red), and vertical bending (orange) at distance *s* from the blade-petiole junctions (**c**). Note that the twisting angle increases continuously, while lateral and vertical bending remain small. Similar trends were seen in three petioles examined (*n* = 3; Fig. S2a–f). **d**–**f** Petiole before lateral light irradiation, analyzed similarly as a negative control, did not show significant bending or twisting (*n* = 3; Fig. S2g–l)

Using the above formulae, we examined Arabidopsis petioles stimulated with lateral blue light irradiation for 3 days (*n* = 3, Figs. 4a–c, S2a–f, Supplementary Movie 2, 3). Spatial changes in twisting angle along the petioles were quantified from the blade-petiole junctions, with the most basal parts excluded, as described above. The twisting was not localized to any part of the petiole (Figs. 4c, S2c, f). Rather, we observed a consistent increase in twisting angle along the entire petiole. This was consistent with the rough observation of a constant rotation of the x–y section of petioles in z-stack images (Supplementary Movie 1). Quantification clearly showed that the twisting angle averaged 15 degrees mm^–1^ (s.d. = 3 degrees mm^–1^) in the observed area. The twisting angle never reversed or dropped to zero in any part of the measured region (Figs. 4c, S2c, f). Petioles without lateral irradiation examined as negative control showed small twisting with average of 2.7 degrees/mm (s.d. = 0.6 degrees mm^–1^) (Figs. 4d–f, S2g–l).

On the other hand, bending in the light-twisted petiole was quite small (< 25 degrees maximum, *n* = 3) in measured regions (Figs. 4c, S2c, f). This was consistent with the stereo-microscopic observation that bending was clearly localized to the most basal part (Fig. 1h, n = 32), which could not be clearly visualized using the light sheet microscope. To summarize, the present measurements clearly showed that bending and twisting have distinct spatial distributions along petioles stimulated by lateral blue light.

## Discussion

Despite more than a century of research, the mechanism underlying stimulus-directed twisting remains unclear (Ambronn 1884; Borchers et al. 2018; Snow 1962). This is partially due to the lack of a measurement method for quantifying local twisting separately from bending. Here, our novel method enabled 3D measurement of twisting along the Arabidopsis petiole. The mathematical definition of a cross section perpendicular to the midline removed any potential bias resulting from bending petioles or inclining petioles relative to the objective lens. This allowed us to determine the angle of twisting between points along the petiole.

Twisting was uniformly distributed along the petiole, in clear contrast to bending, which was localized at the most basal region. The twisting also contrasted to that of pulvinus, a localized motor tissue at the leaf base (e.g. in legumes). This may have resulted from twisting mechanisms differing among plant lineages. Indeed, cell growth is thought to be the primary driver of petiole twisting, whereas pulvinus twisting is driven by local turgor changes (Iino 2001).

What is the difference between the basal region and non-basal regions that contributed to the observed differences in distribution of bending and twisting? Molecular factors such as auxin distribution and cortical microtubule arrangement, which are suggested to be involved in bending and twisting (Buschmann and Borchers, 2020; Ishida et al. 2007; Nakamura and Hashimoto 2020; Smyth 2016; Snow 1962) might be different between the basal region and other parts of the petiole. To check this hypothesis, our method based on data from laser microscopy will enable the monitoring of the distribution of molecules with florescent reporters for comparison with the degrees of deformation, providing insight into the mechanisms underlying petiole movement.

One hypothesized mechanism for twisting is “differential growth of more than two regions” (Ambronn 1884; Dines and Bell 1994; Snow 1962), which is similar to the mechanism of bending (i.e. the “differential growth of two regions”) (Van Overbeek 1939). According to the “more than two regions” hypothesis of twisting, at least three regions on the petiole surface grow at different rates. To resolve this growth difference, more-elongated regions make helices around less-elongated regions, resulting in twisting deformation of the organ. Though exact position of the differentially growing regions is not clear, this hypothesis is consistent with the implied role of the cell growth regulator auxin in stimulus-directed twisting (Borchers et al. 2018; Dines and Bell 1994).

Based on the information on the broad distribution of the light-directed Arabidopsis petiole twisting described in Result section, we estimated the differential growth ratio necessary to produce the experimentally measured angle of petiole twisting. In other words, we estimated the ratio of the length of the least-grown region to that of the most-grown region following the above hypothesis. No bending was considered, as in the real twisting part of the petiole measured above. Least-grown region was assumed to lie in a straight line along the petiole (yellow line in Fig. S1a). Thus, its length was the same as the length of the observed part of the petiole (= L = 3.2 mm). The most-grown region was assumed to be a helix (green curve in Fig. S1a). The radius (= r) of the helix was roughly the radius of the petiole, which was approximately 100 µm. The total angle (= θ) of the helix was set to π/3 rad (= 60°), corresponding to the measured maximum in the previous section. Assuming for simplicity that the helix had a constant angle along the entire petiole, the length of the helix is given as:

$$\sqrt {{\text{L}}^{2} + \left( {{\text{r}}\theta } \right)^{2} } .$$

With the above experimental parameters, the ratio of lengths between the least- and most-grown regions was estimated to be 1.002. This ratio is too small to detect by microscopy. Even when the parameters were artificially set to produce a larger ratio (L = 3 mm, r = 200 µm, and θ = π rad (= 180°)), the ratio was calculated to be 1.01, which is still expected to be too small to detect by microscopy. This might explain scarceness of evidence to support differential growth hypothesis of the twisting.

Automatic 3D segmentation of cells is indispensable for the analysis of petiole deformation at the cellular level. We found that successful segmentation could be achieved for relatively large circular cells using the watershed method with CellAtlas3D on the MorphoGraphX platform (Montenegro-Johnson et al. 2015), whereas thin and/or small cells were mis-segmented (Fig. S1b–e, *n* = 2). This could be improved using other segmentation algorithms (Stegmaier et al. 2018). However, there may be inherent limitations in the observation of cellular morphology and cell size, especially in the most basal part of the petiole and the innermost vasculature, which are difficult to image by microscopy. However, because twisting takes place along the entire petiole, the basal part is not likely to play a mechanically essential role; there are no special structures for transmitting twisting forces along the petiole. The inner region is also unlikely to play an important role because twisting is, by definition, the deformation of outer layers to incline relative to the midline. If one focuses only on outer surface cells, no fixation or optical clearing is necessary, as the outline of an organ can be captured with multi-view fusion in light sheet microscopy. To define the midline without internal imaging, we should first define cross sections as a minimal-area section at each point. Then, the geometric center of gravity of the cross section may be used as the midline. With this modification to omit fixation of plants, our method will be extended to live-imaging to reveal spatiotemporal changes in 3D deformation.

The present system may also be extended to analyze species and organs other than the Arabidopsis petiole we used in this study. The present method depends on tracking the petiole midvein and two margins, taking advantage of the dorsoventrality of petioles. However, even in radially symmetric organs, cell files can be used instead of petiole margins. Thus, our method is not limited to petioles but instead, is applicable to other rod-shaped plant organs that exhibit twisting in various species (e.g. leaf petiole, pulvinus, flower stalk, pedicel, stem, and root) (Buschmann and Borchers 2020; Nakamura and Hashimoto 2020; Smyth 2016). The underlying molecular mechanism might be different as suggested by different trend in chirality (Buschmann and Borchers 2020; Nakamura and Hashimoto 2020; Smyth 2016) and by difference in cell deformation mechanisms (Abraham and Elbaum 2013; Borchers et al. 2018). Applying our method to them, the diversity of flexible plant organ movements will be elucidated.

## Supplementary Information

Below is the link to the electronic supplementary material.

Supplementary Figures S1, 2  (PDF 364 kb)

Supplementary Movie 1. MtrackJ windows are shown from petiole base to blade-petiole junction. As in Fig. 2c, circles #1 indicate the tracked midvein of the petiole, and #2, 3 indicate the tracked margins of the petiole. (MP4 1633 kb)

Supplementary Movie 2. 3D reconstruction of petiole shown in Fig. 4a-c. (MP4 628 kb)

Supplementary Movie 3.3D reconstruction of petiole shown in Fig. 4a-c with yellow balls showing MtrackJ points. (MP4 1091 kb)
