# Correction to: Three-dimensional quantification of twisting in the Arabidopsis petiole

**DOI:** 10.1007/s10265-021-01305-4

**Published:** 2021-04-22

**Authors:** Yuta Otsuka, Hirokazu Tsukaya

**Affiliations:** Department of Biological Sciences, Graduate School of Science, The University of Tokyo, 7-3-1, Hongo, Bunkyo-ku, Tokyo, 113-0033 Japan

## Correction to: Journal of Plant Research 10.1007/s10265-021-01291-7

In the acknowledgment part of the original publication, one of titles was inappropriate. Here is a correct acknowledgment:

We would like to thank Drs. Hiroyuki Takeda, Toru Kawanishi, and Atsuko Shimada (The University of Tokyo, Japan) for allowing us to use the light sheet microscope, Mr. Harunobu Kametani and Ms. Yue Tong (The University of Tokyo, Japan) for assistance in light sheet microscopy, and Dr. Ichiro Terashima (The University of Tokyo, Japan) for helpful suggestions.

