# Supplementary material for: Three-dimensional quantification of twisting in the Arabidopsis petiole
Source: J Plant Res. 2021 Apr 11;134(4):811–9. doi: 10.1007/s10265-021-01291-7 (PMC8245369; doi:10.1007/s10265-021-01291-7)
Supplement: Supplementary file 1 — Supplementary Figures S1, 2 (PDF 364 kb) [file 10265_2021_1291_MOESM1_ESM.pdf]

Electronic supplementary materials

Title:

Three-dimensional quantification of twisting in the Arabidopsis petiole

Authors:

Yuta Otsuka, Hirokazu Tsukaya

Journal:

Journal of Plant Research

Corresponding author:

Hirokazu Tsukaya

(Department of Biological Sciences, Graduate School of Science, The University of Tokyo, Bunkyo-ku, Tokyo, Japan)

Tel: +81-3-5841-4047

Fax: +81-3-5841-4047

E-mail: tsukaya@bs.s.u-tokyo.ac.jp

Content:

Figs. S1–S2

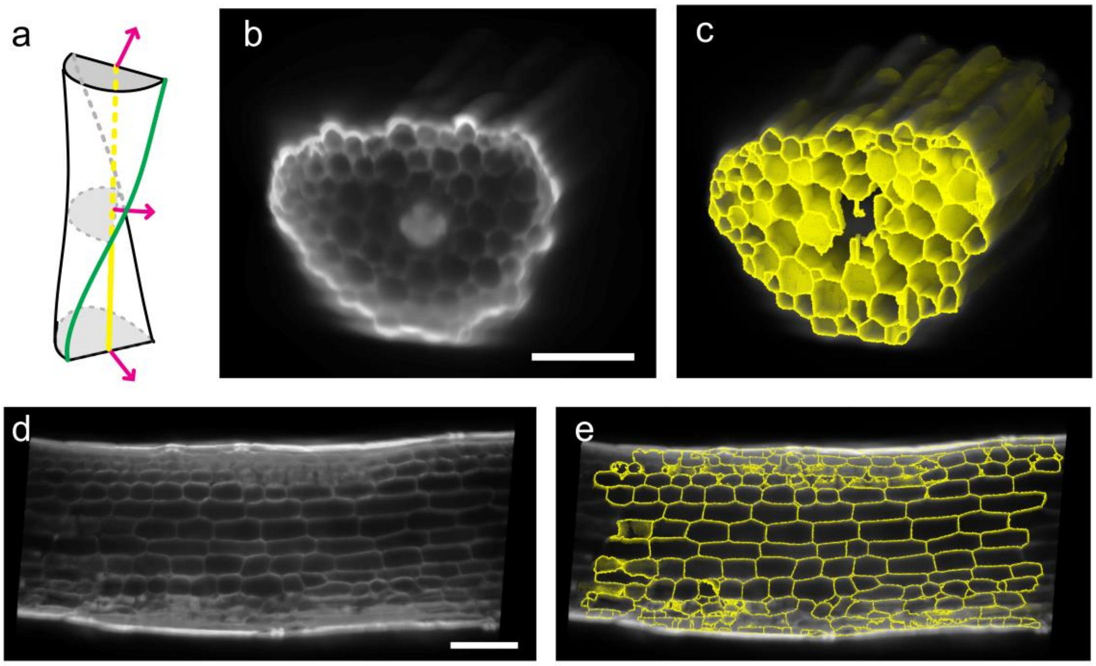

Fig. S1 Small differences in length may result in large degrees of twisting.
(**a**) When differential growth in more than two regions occurs, the shortest region is expected to remain straight (yellow line), while the longest region makes a helix around it with a radius approximately equal to the petiole thickness (green curve). The difference in the length of these two regions was estimated to be small. This calculation would be checked with cell-size measurements from automatic 3D cell segmentation. Watershed segmentation of 3D cells (**c**, **e**) from light sheet microscopy images (**b**, **d**) were shown as the cross section (**b**, **c**) and longitudinal section (**d**, **e**). Note that relatively large cells were successfully segmented, whereas some small cells were mis-segmented. Bars = 100 μm.

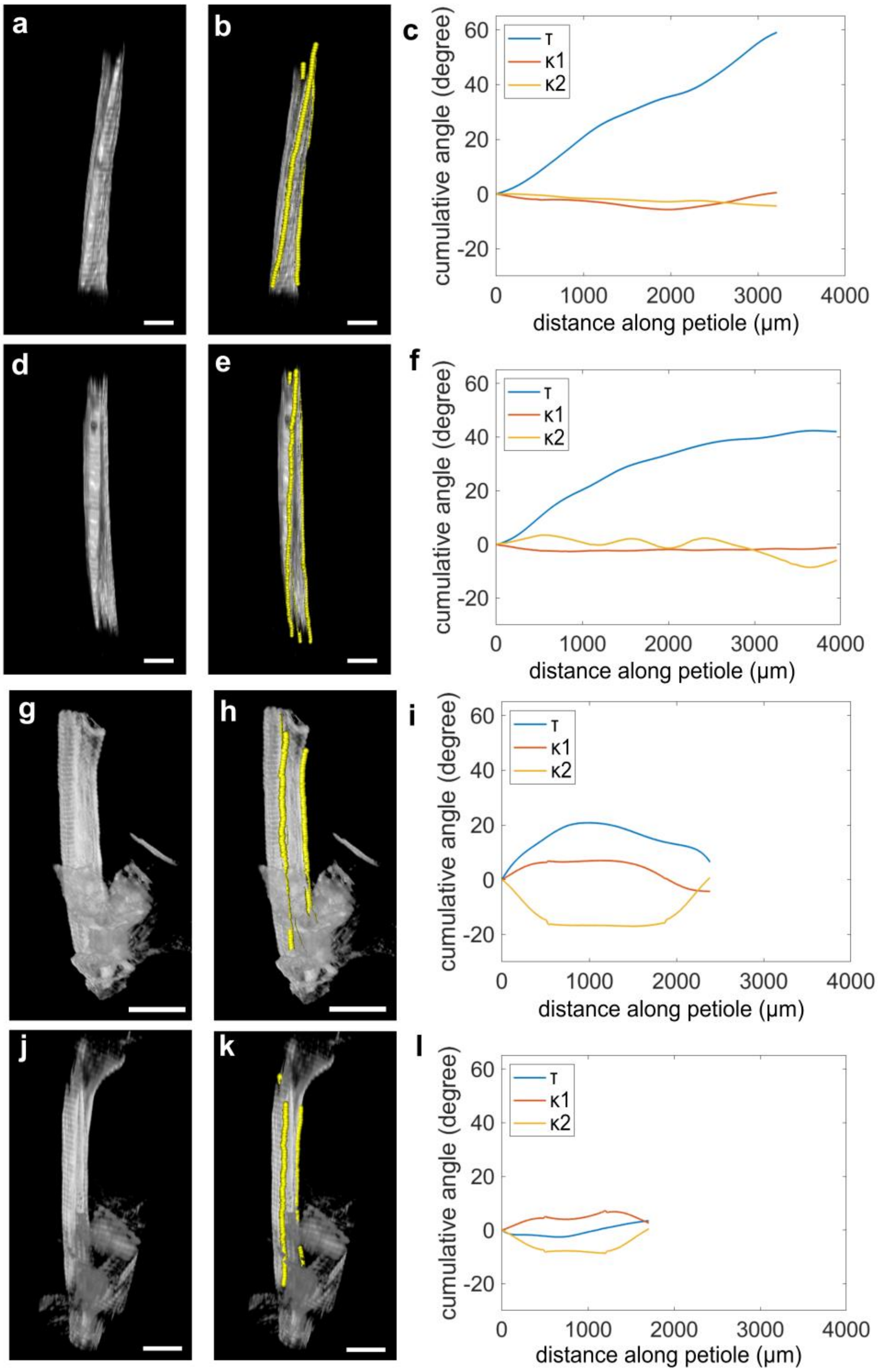

Fig. S2 Additional examples of quantified spatial distribution of twisting and bending.

Additional petioles were examined in similar manner as in Fig. 4. Petiole irradiated with lateral blue light for 3 days are imaged in light sheet microscope (**a**, **d**) and converted to 3D coordinates of midline and margins of petiole shown as yellow balls (**b**, **e**), which were used to calculate cumulative angles of twisting (blue), lateral bending (red), and vertical bending (orange) at distance s from the blade-petiole junctions (**c**, **f**). Petiole before lateral light irradiation were analyzed similarly as a negative control, which did not show significant bending or twisting (**g-l**).
